# Effects of adaptive degrees of trust on coevolution of quantum strategies on scale-free networks

**DOI:** 10.1038/srep02949

**Published:** 2013-10-15

**Authors:** Qiang Li, Minyou Chen, Matjaž Perc, Azhar Iqbal, Derek Abbott

**Affiliations:** 1 State Key Laboratory of Power Transmission Equipment & System Security and New Technology, College of Electrical Engineering, Chongqing University, 400044 Chongqing, China; 2 Faculty of Natural Sciences and Mathematics, University of Maribor, Koroška cesta 160, SI-2000 Maribor, Slovenia; 3 Department of Mathematics and Statistics, King Fahd University of Petroleum and Minerals, 31261 Dhahran, Kingdom of Saudi Arabia; 4 School of Electrical and Electronic Engineering, University of Adelaide, 5005 Adelaide, Australia

## Abstract

We study the impact of adaptive degrees of trust on the evolution of cooperation in the quantum prisoner's dilemma game. In addition to the strategies, links between players are also subject to evolution. Starting with a scale-free interaction network, players adjust trust towards their neighbors based on received payoffs. The latter governs the strategy adoption process, while trust governs the rewiring of links. As soon as the degree of trust towards a neighbor drops to zero, the link is rewired to another randomly chosen player within the network. We find that for small temptations to defect cooperators always dominate, while for intermediate and strong temptations a single quantum strategy is able to outperform all other strategies. In general, reciprocal trust remains within close relationships and favors the dominance of a single strategy. Due to coevolution, the power-law degree distributions transform to Poisson distributions.

Cooperation is surprisingly a commonly observed behavior in human societies as well as in many other biological systems. It is surprising because cooperation means sacrificing individual benefits for the good of others, which inevitably compromises fitness in natural selection. According to Darwin, cooperation should thus die out in a competitive setting. The survival of cooperation has therefore attracted ample attention, and it has indeed become a significant topic studied within the theoretical framework of evolutionary games. From Nowak and May's seminal work^1^ onwards, interesting results^2^,^3^,^4^,^5^,^6^,^7^,^8^,^9^ have been reported using different types of games. In particular, it has been discovered that network structure is a key factor in the evolution of cooperation, which leads to a shift from studying evolutionary games on lattices to studying evolutionary games on complex networks^10^,^11^,^12^,^13^,^14^,^15^,^16^,^17^ (for a review see^18^). Moreover, coevolutionary rules were introduced to evolutionary games, which meant that focus was shifting from the evolution on networks to the evolution *of* networks. For example, the impact of different link-updating rules on the coevolution of strategy and structure^19^,^20^,^21^,^22^,^23^,^24^,^25^,^26^, population growth^27^,^28^, teaching activity^29^,^30^,^31^, as well as the mobility of players^32^,^33^,^34^,^35^,^36^ were studied.

The concept of evolutionary games has also been applied on very small scales, for example to describe interactions of biological molecules^37^,^38^,^39^,^40^, which motivates the consideration of the effects of quantum mechanics^41^. Consequently, a new field of quantum game theory has emerged as the generalization of classical game theory. Notably, several phenomena without classical counterparts have been observed. For example, in the PQ penny flip game^42^, a player who implements a quantum strategy can increase his/her expected payoff. Moreover, when one player is restricted to classical strategies, while the other player is free to adopt quantum strategies, the quantum player can direct the outcome regardless of the classical player's strategy^43^. When the Prisoner's Dilemma game is quantized^44^, the dilemma existing in the classical game is removed, if both players resort to quantum strategies in a restricted space. Moreover, there is a unique Nash equilibrium for the Battle of the Sexes game, if players are allowed to use entangled strategies^45^. It has also been shown that quantum games are more efficient than classical games and a saturated upper bound for this efficiency is found^46^. Further, when the previous performance of agents are considered, evolutionary quantum games^47^ are found to be an appropriate description. If there are a small group of mutants using quantum strategies in the population, the results of how they invade a classical evolutionarily stable strategy (ESS)^48^ and the role of quantum mechanical effects in ESS^49^ have been investigated. Later, quantum repeated games^50^, quantum cooperative games^51^ and quantum correlation games^52^ were studied. Recently, quantum games have also been analyzed by using geometric algebra^53^,^54^,^55^. For further background on quantum games, we refer to^56^,^57^.

If Dawkins' “selfish genes”^58^ are a reality, it may be speculated that games of survival are being played already on the molecular level^44^. In recent years, it has been suggested that quantum mechanics may play a role at the neural level^59^,^60^,^61^. These considerations have provided motivation for investigation of the evolution of quantum and classical strategies on networks using the theory of quantum games. The main difference between a quantum and a classical game is that quantum effects such as entanglement are employed, producing results for which there are no classical counterparts. Moreover, the full quantum strategy space in quantum game theory is a very large space, where the classical strategy set is only a subset of the full quantum space. Also, it is worth noting that a quantum strategy is not a probabilistic sum of pure classical strategies (except under special conditions), and that it cannot be reduced to a set of pure classical strategies^48^.

In this paper, the behavior of agents is described by quantum strategies that are taken from the full quantum strategy space, and agents interact with each other in terms of the model of a quantum game. In accordance with the outcome of interactions, each agent updates its strategy with a given probability and adjusts the degrees of trust that it assigns to neighbors adaptively. As we can observe in reality, people evaluate their friends positively or negatively according to their past behavior, which causes trust in friends to increase or decrease and relationships between them consequently become closer or more estranged. It often happens that low trust also reduces the level of their interactions. Further, if the trust in a friend decreases below a certain value, the relationship between them will be broken and the agent will make a new friend. In the process, reciprocal relationships are often preserved and the degrees of trust between them remain high. Based on this observation, we study how the adaptive degrees of trust influence the coevolution of quantum and classical strategies on heterogeneous networks. After the coevolution comes to an end, new patterns emerge in the population.

The remainder of this paper is organized as follows. First, we present the main results concerning the coevolution on networks, the impact of the adaptive degrees of trust, as well as the resulting degree distributions of nodes after the coevolutionary process reaches a stationary state. Subsequently, we summarize our results and discuss their outreach and potential implications, as well as outline directions for future research. Lastly, in the Methods section, we describe the elementary concepts of a quantum game, in which the classical strategies of cooperation and defection are mapped as two basis vectors in Hilbert space, as shown schematically in Fig. 1. In the Methods section, we also provide a mathematically precise description of the model and the employed coevolutionary rule.

## Results

In this section, the impacts of adaptive degrees of trust on the strategy evolution and the network evolution are investigated. First, the statistical results of the evolution on SF networks are given, and then they are analyzed and explained in detail. Finally, the degree distributions before and after the coevolution are discussed. Before each simulation starts, two quantum strategies are taken from the very large space 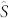 by choosing the parameters, *α*, *β* and *θ* at random, while in all simulations two classical strategies keep the same forms in equation (4). After a simulation, a result including four curves is produced, just like Fig. 2(a)–(b), in which each point on a curve denotes the fraction of agents using a strategy at a given *b*. For the curves corresponding to two quantum strategies, the quantum strategy that produces the topmost curve is defined as *Q*_1_, the other as *Q*_2_. Finally, all results of Monte Carlo simulations are averaged statistically to obtain a statistical result in order to reduce randomness.

Figure 2 exhibits the statistical results of the evolution of four strategies on SF networks. When only the rule of adaptive degrees of trust is introduced into the evolution, but the networks remain static, the results are shown in Fig. 2(a). It can be seen that a quantum strategy is the dominant strategy from the outset. Furthermore, the fraction of agents using the dominant strategy rises slightly with the increase of the temptation *b*. On the other hand, when the coevolution rules described in Methods are employed, there is a chance for cooperators to prevail in the population when *b* is small. However, the strategies, *Q*_1_ and *D*, spread in the population quickly, after the critical value of *b*. Hence, the fractions of agents with these strategies rise significantly, while the fraction of cooperators drops further. Interestingly, defectors' strategies are not imitated by more agents with the rise of *b*, but the fraction always stays at about 20%. It seems that a quantum strategy outperforms the classical ones and consequently the fraction of agents adopting the quantum strategy is far higher than those of others. Furthermore, in all cases cooperators can always survive in the population.

Next, the results of the coevolution are analyzed and explained. As mentioned above, before each simulation, two quantum strategies are chosen randomly from the full quantum strategy space 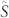. In Monte Carlo simulations, when an agent uses a quantum strategy 
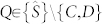
, the cooperation *C* or the defection *D* against a neighbor's strategy 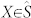, its expected payoff (equation (3)) in each simulation is different, because different quantum strategies are chosen in the simulation. Therefore, the distributions of payoffs over different strategy profiles are calculated at *b* = 1.05 and *b* = 2, as shown in Fig. 3. Based on the statistical analysis, it can be found that most quantum strategists' payoffs are less than one, when *b* is small. With the increase of the temptation *b*, payoffs of agents with a quantum strategy become greater than one gradually, but the probability is still low and most payoffs are approximately one. Furthermore, agents' actual payoffs and total payoffs, as given in equation (6) and equation (5), are closely associated with the adaptive degrees of trust, which leads to a dynamic payoff matrix.

If both agents in a game want to keep their payoffs as high as that without using the adaptive degrees of trust, they need to evaluate the opponent always positively, namely, to keep [*a_t_*(*ij*) + *a_t_*(*ji*)]/2 = 1. However, this happens only if the agent's and its neighbor's behavior or strategies are reciprocal, and the payoff from the opponent is greater than the average of received payoffs. Once an agent evaluates its neighbor negatively, both agents' payoffs will reduce due to [*a_t_*(*ij*) + *a_t_*(*ji*)]/2 < 1, and vice versa. For example, in a *C* − *D* pair, after the first round, the defector evaluates the cooperator positively, whereas the cooperator surely decreases the degree of trust in the defector because its payoff is zero. From the second round on, their payoffs both reduce, because the weight on this link decreases constantly, which can be regarded as punishment for nonreciprocal players. The dynamic payoff influences defectors more than cooperators, because defectors lose their positions as the ones with the highest payoffs. After several rounds, when the defector's actual payoff in the *C* − *D* pair becomes less than the average of all its payoffs, both the defector and the cooperator evaluate each other negatively, which accelerates [*a_t_*(*ij*) + *a_t_*(*ji*)]/2 to become smaller, rather like the Matthew effect. More importantly, this makes the degree of trust decrease to zero faster, and helps the cooperator to break the adverse tie more quickly. Similarly, if there is a *D* − *D* pair, both defectors evaluate the opponent negatively from the first round, because their payoffs are always less than the average. After several rounds, *D* − *D* pairs must be broken in all cases, so do *C* − *D* pairs. When the links are broken, according to the coevolution rules, a cooperator rewires the link in a *C* − *D* pair, while one of defectors in a *D* − *D* pair gets a chance to rewire. Noting that it is not completed in only one operation that the degree of trust decreases to zero. In the process of adjustment, agents get time to know their opponents gradually and give enough chances to them to change their egoistic strategies, which fosters reciprocal strategies to prevail in the population. Moreover, this also makes the time scale for the structural changes be slower than that for the strategy update, which conforms to what we often observe in reality that people change their strategies faster than their relationships between friends.

When the temptation *b* is small, say *b* = 1.05, it is sure that both sides of a *C* − *C* pair evaluate the other positively, because cooperators' payoff, one, are easily greater than the average and their strategies are reciprocal. Thus, *C* − *C* pairs are largely preserved in the population and the degrees of trust between cooperators keep high. As for other pairs, they are preserved or broken, which depends on the degrees of trust in the neighbors. Once the degrees are zeroes, links will be broken. We analyze the weights on all link pairs and the fractions of all link pairs in the population at different time steps, which are depicted in Fig. 4. As shown in Fig. 4(a), the fraction of *C* − *C* pairs soars in the population after about twenty time steps, while others drop largely due to links broken. Consequently, the fraction of *C* − *C* pairs is far higher than others, and the average of weights on *C* − *C* pairs keep the highest too. Hence, as analyzed above, cooperators can accumulate high total payoffs and prevail in the population.

After the temptation *b* exceeds the critical value, the fraction of agents using a quantum strategy rise quickly. When *b* = 2, the fraction reaches the highest in the population. By analyzing the data in Fig. 3(b), we can find that the probability that quantum strategists' payoffs are around one is largest and the probability that they get payoffs greater than one also increases. In this case, it becomes more that the evaluation between quantum strategists is positive. As shown in Fig. 4(c), the fraction of *Q*_1_ − *Q*_1_ pairs is highest, which means that they exist largely in the population. Meanwhile, weights on the links are also quite high. As such, the total payoffs that agents with this strategy collect are higher, which leads the strategy to prevail in the population. The “fates” of *C* − *D* and *D* − *D* pairs are similar to those at *b* = 1.05, i.e., they are broken after several rounds, so their fractions drop largely during the evolution. However, a defector's payoff received from a cooperator is highest and it is twice a cooperator's at *b* = 2. This may make defectors accumulate high total payoffs in a round, so that the strategy *D* also spreads in the population. But the fraction of defectors cannot increase further, for the degrees of trust that the neighbors assign to defectors are lower and lower.

Summing up, in the model, the average of payoffs are used to calculate the probability of imitation, which partly reduces the strong heterogeneity of SF networks. Thus, an agent cannot acquire high total payoffs, if it only counts on the large number of neighbors. More importantly, the adaptive degrees of trust, like punishment, help cooperators to prevail in the population when *b* is small. Furthermore, if the behavior between agents is reciprocal, the degrees of trust in one another can keep high. Therefore, these link pairs are preserved largely in the population. This is why agents on the end of these links can accumulate high total payoffs, which makes the corresponding strategy spread widely in the population and finally become the dominant strategy.

In the last part, the statistical features of networks are also investigated. In our model, links are broken and rewired when the degrees of trust decrease to zero. As such, the network structure evolves over time, and the degree distributions of nodes may change largely. Therefore, we calculate the degree distributions on SF networks after the coevolution at *b* = 1.05 and *b* = 2, which are presented in Fig. 5. In order to obtain more details, we analyze not only the degree distributions of the network, but also that of nodes with the same strategy. Comparing the degree distributions before and after SF networks evolve, we can see that when the rules of the coevolution are employed, the degree distribution of the network totally deviates from the initial power-law distribution after the coevolution and transforms into a Poisson distribution, where the degrees of most nodes in the network are five. Further, it can be observed that the nodes with the largest degree are cooperators at *b* = 1.05, while those are agents with the quantum strategy *Q*_1_ at *b* = 2. Moreover, the largest degree in the network at *b* = 1.05 is a little greater than that at *b* = 2. As is analyzed above, when *b* is small, most agents in the population are cooperators, so that they are connected largely during rewiring, and finally become the nodes with the largest degrees. Similar processes happen at *b* = 2, but in this case, the nodes with the largest degrees are occupied by agents with the quantum strategy *Q*_1_. Meanwhile, defectors are also connected by some agents and acquire high degrees too at *b* = 2, which prevents agents with the quantum strategy *Q*_1_ from acquiring higher degree.

## Discussion

Summarizing, we have proposed a model with coevolutionary rules, in which the adaptive degrees of trust are at the core of the setup. Further, we analyze the impact of adaptive degrees of trust on the evolution of quantum and classical strategies in the quantum prisoner's dilemma game. During the coevolution, instead of only two classical strategies, all agents are allowed to use quantum strategies and play quantized PD games with their immediate neighbors in terms of the model of a quantum game. According to the received payoffs, each agent evaluates its neighbors positively or negatively. In other words, if the payoff from a neighbor is less than the average of all received payoffs of an agent, the degree of trust in the neighbor decreases. On the contrary, if the payoff is greater than the average, the degree of trust increases. Furthermore, agents' total payoffs drop with the decrease of the degrees of trust, which may lower spread of the strategies in the population. Once the degree of trust decreases to zero, the link between an agent and its neighbor is broken and rewired to a randomly chosen agent in the network. Therefore, the adaptive degrees of trust influence the strategy evolution and the network evolution significantly.

We show that when a model without the network evolution is used, a quantum strategy dominates the population from the outset. However, the results based on the model with coevolutionary rules have demonstrated that the strategy *C* is the dominant strategy when *b* is small, while a quantum strategy prevails in the population after *b* is greater than the critical value. As is known, if the behavior between agents is reciprocal, they evaluate each other positively, which results in high degrees of trust between them and preservation of the link pairs. When *b* is small, cooperators' payoffs are easily greater than those of quantum strategists and the averages of received payoffs, so the weights on links keep high and *C* − *C* link pairs exist largely in the population, while others reduce gradually. Consequently, the strategy *C* spreads widely in the population. As *b* rises, the payoffs of quantum strategies increase due to the high degree of trust between them, which ultimately leads to quantum strategy dominance in the population. If *b* = 2, the payoffs of defectors are also high, and hence the *D* strategy is initially imitated by some myopic agents. The initial uprise, however, is quickly dampened because the degree of trust towards the defectors, and thus also the links that connect them with other players, inevitably suffer from their exploitative nature.

In terms of possible directions for further research, influential players could be introduced to the population, and their impact on the coevolution may be investigated. How second-order trust, i.e., the trust a particular individual has in the friend of a friend, influences the coevolution might also be a viable direction to pursue in the future. Furthermore, the framework of quantum evolutionary game theory may be applicable in engineering applications, specifically for the coordination control of distributed generators on a microgrid. If such generators are considered as agents and their behavior is mapped by means of quantum strategies, then their output can be adjusted according to the outcome of the coevolutionary process. Accordingly, a stable and optimal state may be reached gradually during the interaction of agents. In general, it seems safe to conclude that for a broad plethora of classical networks, the currently applied control schemes may be improved and indeed may benefit from the adoption of quantum-like rules at the algorithmic level.

## Methods

### Quantum prisoner's dilemma game

**Figure 1:**
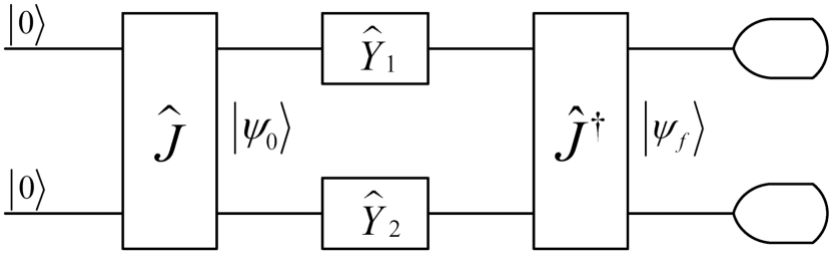
A model of a quantum game. For details on the notation we refer to the Methods section, in particular the subsection Quantum prisoner's dilemma game.

**Figure 2:**
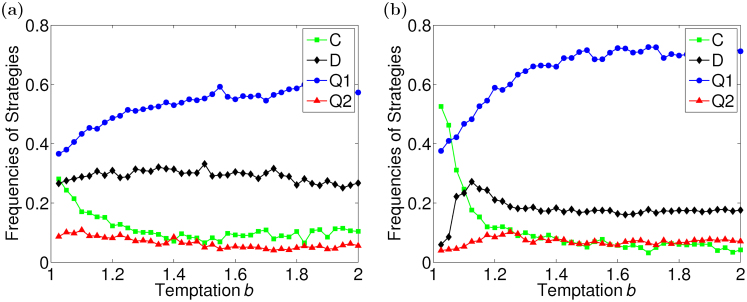
The evolution of strategies as a function of *b*. (a) static networks. (b) dynamic networks. Two panels, (a)–(b), exhibit the fractions of agents using two quantum strategies (*Q*_1_ and *Q*_2_) and two classical strategies (*C* and *D*) in the population after the evolution, where the left panel is obtained when only the rule of adaptive degrees of trust is adopted, but the networks remain static, and the right panel is obtained according to the coevolution rules in Methods. Both panels are statistical results of averaging over 100 simulation results.

**Figure 3:**
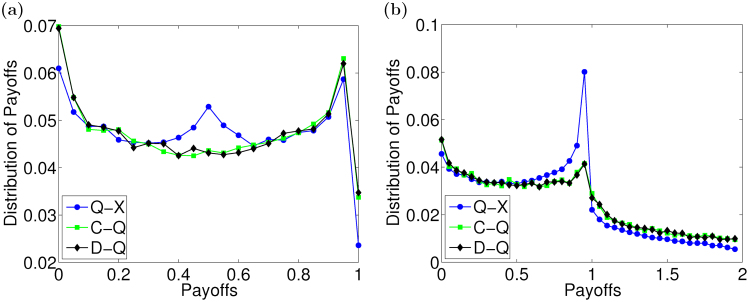
The distributions of expected payoffs over different strategy profiles at different temptation *b*. (a) *b* = 1.05. (b) *b* = 2. First, 2 × 10^4^ quantum strategies are chosen randomly from 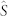, and then payoffs over different strategy profiles are calculated. Finally, the distributions of payoffs are obtained by statistical analysis at different *b*.

**Figure 4:**
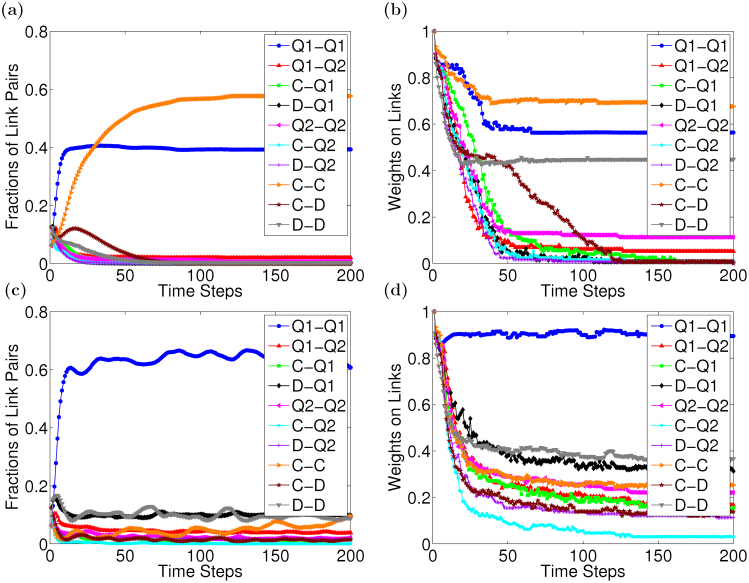
The weights on links and the fractions of link pairs at different temptation *b*. (a) and (c) are the fractions of link pairs. (b) and (d) are the weights on links. (a) and (b) are obtained at *b* = 1.05, while (c) and (d) at *b* = 2. In each simulation, the fractions of all link pairs are calculated at each time step at *b* = 1.05 and *b* = 2, respectively. Meanwhile, for a type of link pairs, e.g. *C* − *C*, all the weights on these links are averaged at each time step at *b* = 1.05 and *b* = 2, respectively. Monte Carlo simulations are carried out over 100 different initial conditions, and then the statistical results are obtained by averaging 100 results of simulations.

**Figure 5:**
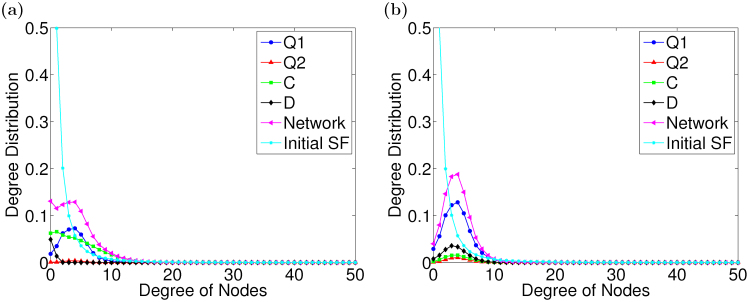
The degree distributions of networks at different temptation *b*. (a) *b* = 1.05. (b) *b* = 2. Two panels, (a)–(b), show the degree distributions separately for different strategies as well as for the network as a whole, which are obtained by averaging those over 100 different initial conditions.

Before describing the coevolutionary model, we briefly introduce the quantized Prisoner's Dilemma (PD) game^44^ first. The classical PD game is widely applied in many scientific fields, where human behavior is abstracted as two strategies, Cooperation (*C*) and Defection (*D*). If both agents use the strategy *C*, they receive Reward (*R*). On the contrary, they get Punishment (*P*) due to both using the strategy *D*. If two agents adopt different strategies, the agent with the strategy *C* receives Sucker (*S*), while the other with the strategy *D* acquires the highest payoff, Temptation (*T*). The focal agent's payoff is given by the following matrix 
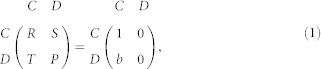
where, without loss of generality, *R* = 1, *T* = *b* (1 < *b* ≤ 2), *P* = 0 and *S* = 0. When the PD game is quantized, the classical strategies, *C* = 0 and *D* = 1, are mapped as two basis vectors {*C* ~ |0〉, *D* ~ |1〉} in Hilbert space and the game is played according to the model of a quantum game, which is shown in Fig. 1^44^.

Assume a quantum game starts in an initial state |00〉, where two qubits belong to two agents. After it goes through a unitary operator 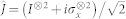, the state is 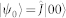. Then, each agent applies its quantum strategy to the qubit, which is chosen from the full quantum unitary strategy space 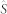
56, 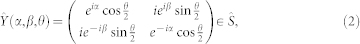
where *α*, *β* ∈ [−*π*, *π*], *θ* ∈ [0, *π*]. Before a projective measurement on the basis {|0〉, |1〉} is performed, the final state is 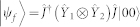. Finally, the focal agent's expected payoff can be calculated as 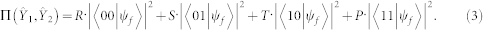


### Mathematical model

Let us consider a finite well-mixed population with *N* = 5000 agents who interact with neighbors by playing quantized PD games, and assume that agents occupy all nodes of a network *G_t_*(*V*, *E*), where *V* is the set of nodes, *E* is the set of links and *t* is the time step. In this paper, a Barabási-Albert scale-free (SF) network^62^,^63^ are considered, in which there are no duplicated links and self loops. The SF network is constructed from a small network with two fully connected nodes, and then a new node with two links is added into the network. The operations of adding new nodes do not stop according to the rules of “growth” and “preferential attachment”, until the total number of nodes is *N*. In the network, the neighbors of an agent *i* are those connected by links between them, so the neighbor set of the agent at a time step *t* can be defined as Γ*_t_*(*i*) = {*j* | *e_t_*(*ij*) ∈ *E*, *j* ∈ *V*\*i*} and the number of neighbors is *k_t_*(*i*) = |Γ*_t_*(*i*)|, where *V*\*i* means the set of nodes, *V*, not including the *i*th node (a complement of {*i*} in *V*) and |·| is the cardinality of a set. Initially, assume each agent in the network totally trusts their immediate neighbors, so the degrees of trust that an agent *i* gives to neighbors are *a_t_*(*ij*) = 1, *j* ∈ Γ*_t_*(*i*). Before each simulation starts, two classical strategies (*C* and *D*) and two quantum strategies are randomly chosen from the full quantum strategy space 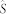. Particularly, the classical strategies, *C* and *D*, have the following forms: 
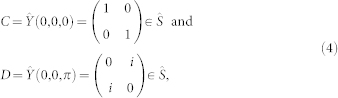
while two quantum strategies, *Q*_1_ and *Q*_2_, are produced by choosing the parameters, *α*, *β* and *θ*, in equation (2) randomly. For example, at the *t*-th simulation, initially, 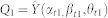, 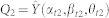. As such, more quantum strategies in the full quantum strategy space rather than two specific quantum strategies can be tested. The four strategies are randomly assigned to all agents with equal probability.

Then, all agents in the network simultaneously play 2 × 2 maximally entangled quantum games with all their neighbors. The total payoff of an agent *i* after the games is the summation of all received payoffs, which can be calculated by 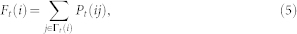

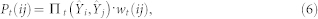

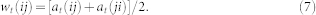
Here, *P_t_*(*ij*) is the actual payoff that the agent obtains and *w_t_*(*ij*) is the average of two degrees of trust, also called a weight on a link, so that *w_t_*(*ij*) = *w_t_*(*ji*) always holds. Subsequently, all agents choose a neighbor from their neighborhood at random. If the total payoff of the selected neighbor is greater than an agent *i*, the agent will imitate this strategy with probability *p_s_*(*i*), which is given by the Fermi rule 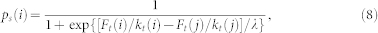
where *λ* is the intensity of selection and set at *λ* = 0.05. Finally, all agents in the network update their strategies synchronously.

Further, according to the payoffs that are received after agents play games with their neighbors, each agent reevaluates its neighbors positively or negatively by increasing or decreasing the degrees of trust. If the payoff from a neighbor *j* is greater than the average of received payoffs, then the neighbor *j* is evaluated positively and the degree of trust is increased by an agent *i*. Otherwise, it is decreased. Therefore, the degree of trust is updated by 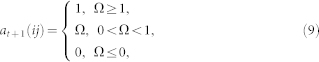

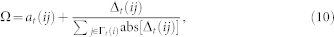

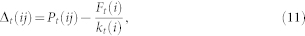
where abs(·) returns the absolute value of a number. It is worth noting that generally the degree of trust that an agent *i* gives to a neighbor *j*, *a_t_*(*ij*), does not equal to that the neighbor gives to the agent, *a_t_*(*ji*), because the payoffs that the agent and the neighbor receive and the adjustment to the degrees of trust are different. If a degree of trust that an agent gives to a neighbor is adjusted negatively several times, it may decrease to zero, which means that the agent does not trust the neighbor any more, so that the link between them is broken. Thus, the agent gets a chance to rewire the link to a randomly chosen agent in the network. Additionally, there exists a particular case that the degrees of trust that both sides of a link give the other are zero. At this time, the agent and the neighbor compete for the chance to rewire. The winner will rewire the link to an agent at random. When the new link is created, the degree of trust that each side gives the other is highest, namely, *a*(*ij*) = *a*(*ji*) = 1. Thereafter, the structure of the network begins to evolve over time.

Monte Carlo simulations are carried out for over 100 times in terms of the above mentioned steps, in each of which the maximum number of iterations is *t* = 10^3^ time steps. After each simulation, the fractions of agents with different strategies are calculated, while the statistical result is obtained by averaging over 100 of simulation results. Particularly, if the variances of the fractions of agents with different strategies are less than 0.01 for 10 consecutive time steps in a simulation after 100 iterations, it is deemed that a steady state has been reached and the iteration ends.

## Author Contributions

Q.L., M.C. and M.P. designed the study; Q.L. and M.P. performed experiments and analyzed the data; Q.L., M.C., M.P., A.I. and D.A. interpreted results and wrote the paper.
